# Ultraviolet radiation exposure and HIV-associated non-Hodgkin lymphoma risk

**DOI:** 10.1186/1750-9378-7-S1-P25

**Published:** 2012-04-19

**Authors:** Shehnaz K Hussain, Otoniel Martínez-Maza, Zuo-Feng Zhang, Lisa Jacobson, Roger Detels

**Affiliations:** 1 Department of Epidemiology, University of California, Los Angeles, CA, USA; 2 Jonsson Comprehensive Cancer Center, University of California, Los Angeles, CA, USA; 3 Department of Obstetrics and Gynecology, David Geffen School of Medicine at UCLA, Los Angeles, CA, USA; 4 Department of Microbiology, Immunology and Molecular Genetics, David Geffen School of Medicine at UCLA, Los Angeles, CA, USA; 5 UCLA AIDS Institute, Los Angeles, CA, USA; 6 Department of Epidemiology, Johns Hopkins University, Baltimore, MD, USA; 7 Division of Infectious Diseases, David Geffen School of Medicine at UCLA, Los Angeles, CA, USA

## Introduction

Although the role of sun exposure to risk of non-Hodgkin lymphoma (NHL) has been controversial, recent studies have suggested a protective effect rather than a promotive effect. The impact of HIV infection on this relationship is unknown, thus we sought to explore the association between sun exposure and sun sensitivity and NHL in the setting of HIV.

## Methods

The study population consisted of a subset of the Multicenter AIDS Cohort Study; 573 HIV+ men who responded to a special ultraviolet radiation exposure questionnaire administered between October 1993 and April 1994, including 33 men who were subsequently diagnosed with pathologically confirmed NHL. The questionnaire elicited information on skin color, natural hair color, eye color, sunburn tendency, average daily sun exposure, occupational sun exposure, recreational sun seeking behaviors, vacationing in sunny locations, sun screen usage, and use of sun lamps or light therapy. Cox proportional hazards regression models were used to obtain hazard ratios (HR) and 95% confidence intervals (CIs) for the association between exposures of interest and NHL risk. HIV positive participants entered the analysis on the date of their UVE questionnaire and were followed until an NHL diagnosis, death, or loss to follow-up. Models were adjusted for race, MACS study site, and CD4+ T cell count.

## Results

Men who reported a high frequency of going to the beach or pool on summer weekends over the last five years had a significantly reduced risk of NHL: HR=0.31 (95% CI =0.15-0.86) for ≥5 times versus never, and HR=0.45 (95% CI=0.20-1.0) for 1-4 times versus never. Compared to men who have rarely taken a beach vacation in the last five years, men who occasionally have were at a significantly reduced risk of NHL (HR = 0.36, 95% CI=0.15-0.86). With respect to sunburn tendency, men who never blister were at reduced risk of NHL compared to men who occasionally blister, although this did not reach statistical significance (HR=0.45, 95% CI=0.19-1.06). Men with green or hazel eyes were at reduced risk of NHL compared to men with blue eyes, although this did not reach statistical significance (HR=0.38, 95% CI= 0.14-1.05).

## Conclusions

Consistent with the NHL literature on HIV uninfected populations, we found that a high level of recreational sun exposure and a low level of sun sensitivity are associated with a decreased risk of NHL in the setting of HIV. Studies are currently underway to elucidate possible mechanisms for these associations, including a possible role of vitamin D.

